# Secondary analysis of change in physical function after exercise intervention in older adults with hyperkyphosis and low physical function

**DOI:** 10.1186/s12877-021-02062-8

**Published:** 2021-02-22

**Authors:** Amy Gladin, Wendy B. Katzman, Yoshimi Fukuoka, Neeta Parimi, Shirley Wong, Nancy E. Lane

**Affiliations:** 1 Chronic Pain Management, San Francisco Kaiser Permanente Medical Center, 4141 Geary Blvd, Suite 212, San Francisco, CA 94118 USA; 2 Department of Physical Therapy and Rehabilitation Science, University of California, San Francisco, 1500 Owens Street, Suite 400, San Francisco, CA 94143 USA; 3 Department of Physiological Nursing, School of Nursing, University of California, San Francisco, 2 Koret Way, San Francisco, CA 94143 USA; 4 California Pacific Medical Center, Research Institute, San Francisco, CA USA; 5 Center for Musculoskeletal Health, University of California at Davis School of Medicine, 4625 2nd Ave, Suite 200, Davis, CA 95817 USA

**Keywords:** Hyperkyphosis, Physical function, Older adults, Health related quality of life

## Abstract

**Background:**

Hyperkyphosis is common in older adults and associated with low physical function and reduced health related quality of life (HrQol). Improved kyphosis has been previously established in kyphosis-targeted interventions in randomized controlled trials in older adults with hyperkyphosis; however, evidence for improved physical function is conflicting. Few studies have investigated change in physical function after a targeted kyphosis intervention in older adults with low physical function. The primary aim in this descriptive study was to explore change in physical function after a progressive high-intensity 3-month targeted kyphosis exercise and posture training intervention in older adults with low physical function and hyperkyphosis. Secondary aims were to explore change in HrQol, spinal strength and spinal curvature, and adherence and safety of the intervention.

**Methods:**

In this secondary analysis of the Specialized Center of Research (SCOR) Kyphosis randomized trial, 101 community dwelling older men and women with hyperkyphosis who completed the intervention were divided into a low function group (LFG) and high function group (HFG). Baseline characteristics were compared between LFG and HFG. Physical function, HrQol, spinal strength and spinal curvature (kyphosis and lordosis) pre/post intervention change scores were explored within and between groups. Adherence and adverse events were examined in the LFG and HFG.

**Results:**

Twenty-six (26%) older adults were LFG, mean Short Phyiscal Performance Battery (SPPB) 9.62 (SD = 1.17) points. At baseline, the LFG was older than HFG (*p* = 0.005), experienced more pain, (*p* = 0.060), had worse physical function and HrQol (*p* ≤ 0.001), and comparable kyphosis (*p* = 0.640). SPPB changed 0.62 (95% CI: − 0.20 to 1.44) points in the LFG and - 0.04 (95%CI: − 0.28 to 0.19) points in the HFG, *p* = 0.020. Gait speed changed 0.04 (95% CI: − 0.02 to 0.10) m/s in the LFG. Kyphosis improved equally in both groups. Adherence to the intervention was 83% in the LFG and 79% in the HFG. There were no adverse events in either group.

**Conclusions:**

Older adults with low physical function and hyperkyphosis may improve physical function after a kyphosis targeted intervention. Older adults with low physical function may safely participate in targeted high-intensity kyphosis exercise and posture training. This observation needs to be confirmed in larger adequately powered studies.

**Trial registration:**

Clinicaltrials.gov identifier: NCT01766674.

**Supplementary Information:**

The online version contains supplementary material available at 10.1186/s12877-021-02062-8.

## Introduction

Age-related hyperkyphosis, commonly defined by a thoracic spine Cobb angle curvature of 40 degrees or greater, progresses with age and affects up to 40% of older adults [1]. Hyperkyphosis in older adults has been associated with impaired physical function, reduced health-related quality of life (HrQol), increased falls and fracture risk and is a predictor of all-cause mortality in women [1–10]. Numerous cross-sectional and longitudinal studies have demonstrated hyperkyphosis is associated with slowed gait speed cross-sectionally and is predictive of worsening chair-stand time and Timed Up and Go (time to rise from chair, walk 10-m turn and return to sit) performance longitudinally in adjusted models [2–6, 11]. Furthermore, hyperkyphosis has been identified as a ‘new’ geriatric syndrome, thus targeting hyperkyphosis as an impairment may contribute to slowing the progression to physical frailty [12].

It is theorized that hyperkyphosis causes an anterior displacement of the center of gravity, which affects physical function characteristics and balance, and in turn negatively impacts physical function [13]. Therefore, it has been hypothesized that interventions that reduce kyphosis may also improve physical function; however, few randomized controlled trials targeting improvement of kyphosis have reported a significant increase in physical function despite successfully reducing kyphosis [14–18]. Previous trial results could be explained by a ceiling effect in high functioning cohorts of individuals who were already functioning above age-matched normative values for physical function. Further investigation of the effects of a targeted kyphosis intervention on physical function in older adults with low function and hyperkyphosis may inform future treatment for older adults at risk for physical function decline and frailty.

Since it is unknown if older adults with hyperkyphosis and low physical function who undergo a targeted kyphosis intervention will also improve physical function, we performed a secondary analysis using data from the Specialized Center of Research (SCOR) randomized controlled trial that investigated change in kyphosis, physical function and HrQoL in older adults with hyperkyphosis after a 3-month targeted high-intensity kyphosis exercise and posture training intervention [14]. We categorized participants post-hoc into low and high physical function groups according to baseline physical function scores. We hypothesized that older adults with hyperkyphosis, low physical function, and who are transitioning to frailty would improve physical function after a targeted kyphosis intervention. Our primary aim was to explore change in physical function in older adults with hyperkyphosis and low physical function, and whether they responded differently than adults with higher function. We also explored change in HrQol, spinal strength and spinal curvature in low versus high function groups. Lastly, we explored the feasibility of conducting a targeted high-intensity kyphosis intervention in older adults with hyperkyphosis and low physical function by comparing adherence to the intervention and safety in both low and high function groups. We used change in physical function scores in the low function group to determine a sample size needed to test the hypothesis in a future, fully powered trial.

## Methods

### Study design

This secondary data analysis included participants (*n* = 101) who completed the SCOR kyphosis intervention in a randomized controlled waitlist design trial [14]. In the SCOR trial, between group comparisons were made between the active and control group at 3-months, then the waitlist group received the intervention and a 6-month assessment to investigate within subject change after receiving the intervention for all participants. For the secondary analysis, we calculated a baseline Short Physical Performance Battery (SPPB) score post-hoc using subcomponents of baseline measurements to divide the SCOR cohort into a low functioning group (LFG) and a high functioning group (HFG). The SPPB is a lower extremity strength, mobility and balance physical performance measure, and a composite measure of gait speed, Five-times Sit to Stand and ability to stand with feet together, feet in half tandem and feet in full tandem for 10 s for each condition [19]. Each component is scored on an ordinal scale from 0 to 4, where 0 represents lowest ability and 4 represents highest ability, with a maximum score of 12 possible points. The SPPB is predictive of future mobility decline and incident activities of daily living disability [19–21]. The definition of low physical function was operationalized by an established cut-off score of 10 or less on the SPPB that identifies older adults who are at-risk for mobility decline [20, 22]. A score of 10 or less has been identified as the best cut-off point for the determination of the physical frailty process with a likelihood radio of 1.59 [20, 22]. An SPPB score of 11 or 12 was categorized as the HFG. The SPPB has high levels of reliability, good to moderate concurrent validity with quality of life, strength, muscle power and mobility and scores less than 10 are predictive of all-cause mortality [23, 24].

### Study participants

Participants in the original SCOR trial (*n* = 112) were recruited from a university-based medical center and an integrated managed-care center in San Francisco from January 2013 to June 2015 and included community-dwelling adults age 60 or greater with hyperkyphosis > 40 degrees, English language proficient, able to walk 1 block without an assistive device and rise from a chair without their hands [14]. Participants were excluded from the SCOR trial if they were unable to actively extend their thoracic spine by at least 5 degrees or had cognitive impairment [25]. Participants were initially randomized to an active (*n* = 57) or waitlist control (*n* = 55) group; however, 9 withdrew within the first week due to lack of time or interest and 2 did not have analyzable baseline radiographs for Cobb angle measurements. Participants (*n* = 101) who completed the trial were included in the secondary analysis. The trial protocol was approved by the Institutional Review Boards at the University of California, San Francisco and Kaiser Permanente Northern California. Written informed consent was obtained from all participants. The study protocol and methods were performed in accordance with the guidelines and regulations of the Declaration of Helsinki.

### Intervention groups

The SCOR active intervention was a physical therapist led group targeted kyphosis exercise and posture training program for 1-h twice weekly for 3 months [14]. A licensed physical therapist led the intervention and provided participants with verbal feedback and a trained research assistant provided additional supervision and feedback. A ratio of 5 study participants to 1 staff member was maintained during every intervention session. The active intervention included progressive high-intensity spinal and lower extremity strengthening exercise, thoracic spine and lower extremity range of motion exercise, and posture training. Participants were asked to practice good posture during activities of daily living outside the intervention and provided with an educational handout with pictures to reinforce good posture during activities of daily living. Details on the exercise and posture training intervention have been previously published [26]. A wait-list control group received usual care during the initial 3-months and received the active intervention after the 3-month waitlist period, thus all participants received the intervention.

### Demographic and other measures

Prior to randomization in the original SCOR trial, participants provided demographic and health information via self-report (age, sex, education, co-morbidities). Height and weight were collected using standard measures, and body mass index was calculated. Bone mineral density of the hip and spine was measured using dual-energy X-ray absorptiometry (GE Lunar Prodigy, Madison, WI, USA). Baseline standing lateral spine radiographs were were performed at a University radiology clinic and followed a standardized protocol for measurement of standing lateral spine [27]. Participants were evaluated for prevalent vertebral fractures and diffuse idiopathic skeletal hyperostosis (DISH) by experienced assessors [28–30].

### Outcome measures

SCOR study outcome measures were performed at baseline, 3-month and 6-month timepoints (for the waitlist group only). The waitlist group received the intervention after the 3-month measurement. All measurements were performed by trained examiners masked to group allocation (no waitlist, waitlist).

### Physical function

Performance-based physical function was assessed using the modified Physical Performance Test, 4-m walk test, Timed Up and Go (TUG) and 6 min Walk (6 MW) tests [31–33]. The modified Physical Performance Test included 7 standardized timed tasks: 50-ft floor walk, donning and doffing a lab coat, picking up penny from floor, Five-times Sit to Stand test from a 41 cm chair without using upper extremities, lifting a 7-pound book, climbing one flight of stairs, standing balance and two untimed tasks: climb up/down 4 flights of stairs and performing a 360 degree turn [33]. Each component is scored 0 to 4 with a maximum score of 36. Scores 25 to 31 indicate mild frailty [34]. The 4-m walk test (gait-speed) measures time to walk 4 m (meter/second) [31]. The 6 MW test measures distance (meters) walked in 6 min [31]. The TUG measures time(s) to rise from a chair, walk 10-m turn and return seated to the chair [31]. The four-meter walk test, 6 MW and TUG are well described in the literature and have good to excellent reliability among older adults with arthritis [31, 32]. The Patient-Reported Outcome Measuremement Information System (PROMIS) Physical Function questionnaire is scored using a t-score metric and is calibrated to have the population mean be a t-score of 50 with the standard deviation set to be 10 [35]. Scores range from 0 to 100 and higher t-scores indicate improved physical function. The SPPB score was calculated post-hoc from sub-components of the modified Physical Performance Test and 4-m walk test data.

### Health Related Quality of Life (HrQol)

Participants completed a battery of patient reported HRQoL outcomes including, PROMIS Global Health Scale v.1.0 (both physical and mental health individual scores), the modified Scoliosis Research Society 30, self-image domain and the Physical Activity Scale for the Elderly (PASE) [36–38]. For all HrQol measures, an increase in score indicates an improvement within the specific domain (see Table 2 for score ranges). Pain and self-rated general health outcomes were extracted from single questions within the PROMIS Scale v.1.0 - Global Health [39]. The pain measurement within the PROMIS Global Health questionnaire utilizes a visual analogue scale and states ‘In the past 7 days, how would you rate your pain on average’ 0 indicates ‘no pain’ and 10 indicates ‘worst imaginable pain’.

### Spinal strength

Spinal strength was measured using the Biodex 3 (Biodex Medical Systems Inc., Shirley, NY, USA), a computerized dynamometer for spinal flexors and spinal extensors [40]. Spinal endurance was measured with the Timed Loaded Standing test**,** a combined measure of trunk and arm endurance, and is quantified as the time in seconds able to hold a 2-pound dumbbell in each hand with both shoulders flexed to 90 degrees and elbows extended to neutral [41].

### Spinal curvature and cobb angle kyphosis

**Fig. 1:**
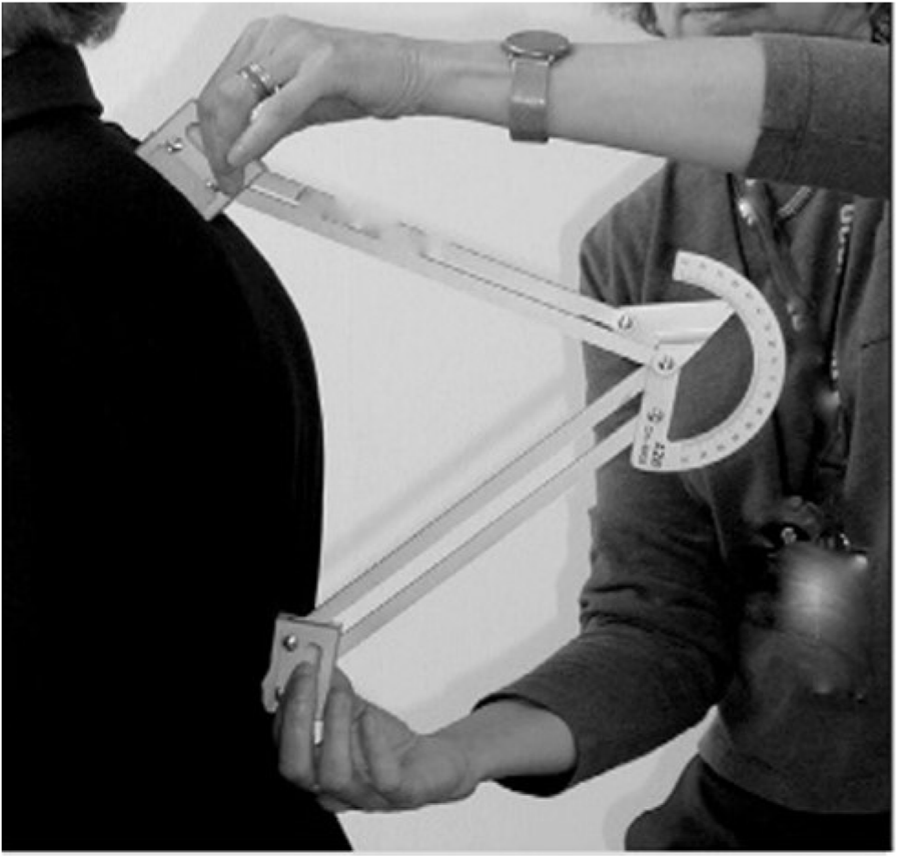
Debrunner Kyphometer. The Debrunner Kyphometer measures an external Cobb angle with the feet of the kyphometer placed over the T2/3 and T11/12 interspaces

Clinical measures of thoracic kyphosis and lumbar lordosis were measured in a usual standing posture with the Debrunner Kyphometer (Techmedica Inc., Camarillo, CA, USA) [42, 43]. The Debrunner Kyphometer measures an external Cobb angle with the feet of the kyphometer placed over the T2/3 and T11/12 interspaces (Fig. 1). Cobb angle of kyphosis measurements were made from standing lateral spine radiographs according to standardized protocols by an experienced radiologist [27].

### Adherence and safety

Adherence to the intervention sessions, adverse events and non-reportable events were monitored by the study coordinator, who administered a standardized questionnaire on a weekly basis during the active intervention. Mean adherence to sessions was calculated as a percentage based upon the number of sessions attended out of the 24 sessions possible. Adverse events are defined as any unfavorable medical occurrence and problems that are possibly related to study participation serious or unexpected. Non-reportable events are expected symptoms that may occur during the intervention and described in the study protocol and disclosed in the consent. Examples of non-reportable events in this study are muscle or joint soreness or exacerbation of previous injuries associated with the intervention and resolved within an expected duration of time [44].

### Data analysis for secondary analysis

Baseline characteristics were compared between the LFG and HFG using t-tests or Wilcoxon nonparametric tests for continuous variables and the χ^2^ statistic for categorical variables. We calculated mean differences and 95% confidence intervals for change pre/post treatment unadjusted for any covariates for all outcome measures in the LFG and HFG. We compared the pre/post intervention change scores in the LFG and HFG using t-tests. In exploratory analysis we performed a sensitivity analysis and adjusted for age and gender. *P*-values < 0.05 were considered statistically significant. Analyses were conducted using SAS Version 9.4 (SAS Inc., Cary, NC). In this post-hoc secondary analysis, we did not have power to detect significant differences within the groups based upon the small sample size. We determined sample sizes needed for a future study to test the hypothesis that older adults with hyperkyphosis and low physical function will improve physical function on the SPPB after a kyphosis exercise and posture training intervention. Sample size calculations were conducted using PASS 15.0.3 software (Power Analysis & Sample Size, NCSS Software, Kaysville, UT, USA).

## Results

There were more men in the LFG versus HFG, 54% vs 36%, *p* = 0.110. The LFG was older than the HFG, 72.4 (SD = 6.5) years versus 68.8 (SD = 5.15) years, *p* = 0.005, and reported poorer general health, *p* = 0.006, and more pain 3.3 (SD = 2.7) points in the HFG and 2.0 (SD = 1.7) in the LFG, *p* = 0.060, (Table 1).

**Table 1:** Study participant characteristics at baseline in the HFG and LFG

Variable	Category	Low Function Group (LFG) *n* = 26	High Function Group (HFG) *n* = 75	LFG vs. HFG*	Entire Cohort *n* = 101
		N (%), mean ± SD	N (%), mean ± SD	*p*-value	N (%), mean ± SD
Age (years)		72.4 ± 6.6	68.8 ± 5.2	0.005	69.7 ± 5.7
Gender	Female	12 (46)	48 (64)	0.110	60 (59)
Vertebral Fracture	none	22 (85)	65 (87)	0.741	87 (86)
	1	2 (8)	7 (9)		9 (9)
	2	2 (8)	3 (4)		5 (4.95)
Diffuse idiopathic hyperostosis (DISH) present (yes)		7 (28)	15 (21)	0.461	22 (23)
Body Mass Index(kilograms/meter^2^)		27.7 ± 3.9	25.7 ± 4.1	0.037	26.2 ± 4.1
Bone mineral density total hip t-score		−0.5 ± 1.3	−0.9 ± 1	0.074	−0.85 ± 1.1
Bone mineral density total spine t-score		0.7 ± 2.8	−0.5 ± 1.8	0.064	−0.2 ± 2.2
Race	Caucasian	25 (96)	69 (92)	0.472	94 (93)
Education	High school, some College	2 (8)	11 (15)	0.360	13 (13)
	College, professional degree	24 (92)	64 (85)	0.360	88 (87)
Pain Score from PROMIS scale 1.0-Global health, 0–10 (points)		3.3 ± 2.7	2 ± 1.7	0.060	2.3 ± 2.1
Self-rated health from PROMIS scale 1.0-Global Health	Fair	5 (19)	3 (4)	0.006	8 (9)
	Good	12 (46)	19 (25)		31 (31)
	Very Good	7 (27)	41 (55)		48 (48)
	Excellent	2 (8)	12 (16)		14 (14)
Co-morbidities	2 or more	10 (38)	30 (40)	0.890	40 (40)

*SD* standard deviation, *PROMIS* Patient-Reported Outcome Measurement Information System, *LFG* low functioning group, *HFG* high functioning group, *p values for comparison between LFG and HFG

Twenty-six percent (*n* = 26) adults had low function, mean SPPB score 9.62 (SD = 1.2) points, and 74% (*n* = 75) were considered high functioning, mean SPPB score 11.37 (SD = 0.7) points (Table 2). At baseline, all physical function and HrQol scores were lower in the LFG versus the HGF, *p* = 0.017, except physical activity was similar in both groups, *p* = 0.330. Trunk endurance was worse in the LFG, *p* = 0.017. There were no differences in baseline levels of kyphosis, *p* = 0.216.

**Table 2:** Means of measures at baseline stratified by HFG, LFG and entire cohort

Outcome measures	Low Function Group (LFG) *n* = 26	High Function Group (HFG) *n* = 75	LFG vs. HFG*	Entire cohort *n* = 101
	Mean (±SD)	Mean (±SD)	*P*-value	Mean (±SD)
**Physical Function**				
Short Physical Performance Battery (SPPB) (0–12 points)	9.62 (1.2)	11.37 (0.7)	< 0.001	10.92 (1.1)
4-m gait speed (meters/second)	1.14 (0.2)	1.38 (0.34)	< 0.001	1.32 (0.3)
Modified PPT (0–36 points)	31.4 (2.7)	33.8 (1.7)	< 0.001	33.2 (2.2)
Timed Up and Go (seconds)	8.4 (1.6)	7.1 (1.5)	< 0.001	7.4 (1.7)
Six Minute Walk Test (meters)	471.7 (77.2)	524.9 (95.9)	0.008	511.6 (94.1)
PROMIS Physical function t-score (0–100)	44.8 (4.7)	50.1 (7.6)	< 0.001	48.7 (7.3)
**Health-related Quality of Life**				
SRS 30 Self-image (0–5 points)	3.33 (0.49)	3.69 (0.56)	0.016	3.53 (0.55)
PROMIS Global Health Scale (0–100)	35.8 (6.5)	40.7 (4.7)	0.001	39.5 (5.6)
PROMIS Global Health Scale, Mental Health t-score (0–100)	48.8 (8.1)	54.1 (7.5)	0.003	52.8 (7.9)
PROMIS Global Health Scale, Physical Health t-score (0–100)	47.9 (7.5)	53.6 (5.6)	< 0.001	52.2 (6.6)
PASE activity level (0–793) points	102.1 (60.5)	111.0 (53.2)	0.330	108.7 (54.9)
Pain Score 0–10 from PROMIS Global Health scale (0–10 points)	3.3 (2.7)	2.0 (1.68)	0.060	3.4 (2.1)
**Spinal Strength**				
Spinal flexion strength (percent peak torque/body weight)	28.8 (11.4)	32.7 (11.3)	0.119	31.7 (11.4)
Spinal extensor strength (percent peak torque/body weight)	64.2 (16.2)	71.0 (22.0)	0.238	69.2 (20.8)
Timed Loaded Standing (seconds)	112.0 (49.9)	138.4 (49.0)	0.017	131.0 (50.3)
**Spinal Curvature**				
Cobb angle of kyphosis (degrees)	56.8 (13.3)	55.5 (11.7)	0.640	55.9 (12.2)
Kyphosis derived from kyphometer (degrees)	53.6 (6.2)	51.5 (7.8)	0.216	52.0 (7.4)
Lordosis derived from kyphometer (degrees)	25.9 (12.2)	31.7 (11.4)	0.028	30.2 (11.8)

*SD* Standard Deviation, *Modified PPT* Physical Performance Test, *SRS* Scoliosis Research Society, *PASE* Physical Activity Scale for the Elderly, *PROMIS* Patient-Reported Outcome Measurement Information System, *HFG* high functioning group, *LFG* low functioning group, group *p values for comparison between LFG and HFG

### Within group change in physical function in low function group

There were no significant within-group changes in SPPB, 4-m gait speed, modified Physical Performance Test, TUG, 6 MW or PROMIS physical function in the LFG in the unadjusted analysis (Table 3). The mean SPPB score changes for the LFG was 0.62 (95%CI: − 0.2 to 1.44) points and 4-m gait speed mean change was 0.04 (95%CI: − 0.02 to 0.10) m/s. TUG mean change was 0.2 (95%CI: − 0.6 to 0.3) seconds and the 6 MW mean change was 1.8 (95%CI: − 14.0 to 17.6) meters. After controlling for age, the LFG (mean SPPB = 9.6) improved 0.77 (95%CI: 0.23 to 1.3) points (Additional file 1). After controlling for age and sex, improvement in SPPB increased to 0.84 (95%CI: 0.31 to 1.38) points (Additional file 2). After controlling for age and age and sex, there were no significant within-group changes in the LFG in 4-m gait speed, modified Physical Performance Test, TUG, 6 MW or PROMIS physical function.

**Table 3:** Change scores pre/post intervention in outcome measures in the HFG, LFG and overall cohort with confidence intervals (CI)

	Low -Function group (LFG)	High-Function group (HFG)	LFG vs HFG*	Entire cohort
	Mean difference (95% CI)	Mean difference (95% CI)	*p*-value	Mean (95% CI)
**Physical Function**				
Short Physical Performance Battery (SPPB) (0–12 points)	0.62^a^ (− 0.20 to 1.44)	− 0.04 (− 0.28 to 0.19)	0.020	0.13 (− 0.14 to 0.40)
4-m gait speed (m/s)	0.04^a^ (− 0.02 to 0.1)	−0.01 (− 0.06 to 0.04)	0.406	0 (− 0.04 to 0.04)
Modified PPT (0–36 points)	−0.29 (− 2.7 to 2.1)	−1.3 (− 3.0 to 0.3)	0.166	−1.1 (− 2.4 to 0.3)
Time up and go (seconds)	−0.2 (− 0.6 to 0.3)	0.04 (− 0.2 to 0.3)	0.370	−0.01 (− 0.2 to 02)
6 Minute walking test (meters)	1.8 (− 14.0 to 17.6)	8.8 (− 4.6 to 22.1)	0.265	7.0 (−3.6 to 17.6)
PROMIS Physical Function t-score (0–100 points)	0.91 (− 5.5 to 2.3)	2.0 (0.5 to 3.5)	0.353	1.7 (0.5 to 2.9)
**Health-related Quality of Life**				
SRS 30 Self-image (0–5 pts)	0.19 (0.02 to 0.4)	0.24 (0.1 to 0.3)	0.473	0.23 (0.1 to 0.3)
PROMIS Global Health Scale (0–100 points)	1.0 (− 0.8 to 2.8)	0.3 (− 0.5 to 1.1)	0.820	0.5 (− 0.2 to 1.2)
PROMIS Global Health scale, Mental Health t-score (0–100 points)	1.19 (− 0.8 to 3.2)	0.3 (− 0.9 to 1.5)	0.536	0.52 (− 0.5 to 1.6)
PROMIS Global Health scale, Physical Health t-score (0–100 points)	1.8 (− 0.3 to 3.9)	0.6 (− 0.5 to 1.8)	0.194	0.9 (− 0.06 to 1.9)
PASE activity (0–793) points	6.3 (− 14.0 to 26.6)	1.7 (− 8.2 to 11.6)	0.928	2.9 (− 5.9 to 11.7)
Pain Score 0–10 from PROMIS Global Health scale (0–10 points)	− 0.2 (− 0.9 to 0.5)	−0.3 (− 0.6 to 0.06)	0.54	−0.26 (− 0. 5 to 0.03)
**Spinal Strength**				
Spinal Flexion (percent peak torque/bodyweight)	−0.09 (− 3.4 to 3.2)	1.5 (− 0.1 to 3.2)	0.515	1.12 (− 0.4 to 2.6)
Spinal extensor (percent peak torque/bodyweight)	−6.8 (− 16.1 to 2.6)	6.0 (1.0 to 11.0)	0.018	2.7 (− 1.8 to 7.2)
Time loaded standing (seconds)	−8.8 (− 23.3 to 5.7)	5.2 (− 1.6 to 12)	0.080	1.6 (− 4.7 to 7.8)
**Spinal Curvature**				
Cobb Angle (degrees)	−0.6 (− 2.0 to 0.9)	− 1.6 (− 2.7 to − 0.4)	0.390	− 1.3 (− 2.2 to − 0.4)
Kyphosis (degrees)	−3.1^a^ (− 5.2 to − 0.9)	−3.7^a^ (− 5.0 to − 2.4)	0.409	−3.5 (− 4.6 to − 2.4)
Lordosis (degrees)	0.4 (−2.0 to 2.9)	− 1.5 (− 3.0 to 0.03)	0.229	−1.0 (− 2.3 to 0.3)

*CI* confidence interval, *Modified PPT* Physical Performance Test, *SRS* Scoliosis Research Society, *PASE* Physical Activity Scale for the Elderly, *HFG* high functioning group, *LFG* low functioning group

^a^denotes change scores surpassing minimum clinical change estimates (SPPB .03 to .08 points and gait speed .03 to .06 m/s and kyphosis Minimum Detectable Change 2.51 degrees) [18, 45, 46]

*p values for comparison between LFG and HFG

### Within group change in HrQol, spinal strength and spinal curvature

Self-image, a measure of HrQol, improved within both groups 0.19 (95%CI: 0.02 to 0.4) points in the LFG and 0.24 (95%CI: 0.1 to 0.3) points in the HFG (Table 3). Spinal extensor strength had a mean change score of - 6.8 (95%CI: − 16.12 to 2.56) percent in LFG and improved 6.0 (95%CI: 1.0 to 11.0) percent in the HFG. Spinal endurance measured by the Timed Loaded Standing mean change was − 8.8 (95%CI: − 23.3 to 5.7) seconds in the LFG and 5.2 (95%CI: − 1.6 to 12) seconds in the HFG. Kyphometer measured kyphosis improved − 3.1 (95%CI: − 5.2 to − 0.9) degrees and − 3.7 (95%CI: − 5.0 to − 2.4) degrees in the LFG and HFG respectively. PROMIS Global health, physical health scale improved 2.3 (95%CI: 0.25 to 4.39) points in the age and sex adjusted model in the LFG and 2.2 (95%CI: 0.15 to 4.21) points in the age adjusted model in the LFG (Additional files 1 and 2).

### Between group change in physical function in low function versus high function

Comparing change pre/post intervention in the LFG and HFG, the LFG improved SPPB more than the HFG, *p* = 0.020 (Table 3). The mean SPPB score changes for the LFG was 0.62 (95%CI: − 0.2 to 1.44) points and − 0.04 (95%CI: -0.02 to 0.10) m/s in the LFG and -0.01 (95%CI: -0.06 to 0.04) m/s in the HFG, *p*=0.406), TUG, (0.2 (95%CI: -0.6 to 0.3) seconds in the LFG and 0.04 (95%CI: -0.2 to 0.3) seconds in the HFG, *p*=0.370), or 6MW, (1.8 (95%CI: -14.0 to 17.6) meters in the LFG and 8.8 (95%CI: − 4.6 to 22.1) meters in the HFG, *p* = 0.265) in the unadjusted analysis (Table 3). Comparing change pre/post intervention in the LFG and HFG, the LFG improved SPPB more than the HFG in the age-adjusted model, *p* = 0.008 and the age and sex adjusted model, *p* = 0.003 (Additional files 1 and 2). After controlling for age, the LFG (mean SPPB = 9.6) improved 0.77 (95%CI: 0.23 to 1.3) points while the HFG (mean SPPB = 11.4 points) changed - 0.093 (95%CI: − 0.04 to 0.22) points (Additional file 1). After controlling for age and sex, improvement in SPPB increased further to 0.84 (95%CI: 0.31 to 1.38) points and changed − 0.12 (95%CI: − 0.43 to 0.19) points in the HFG (Additional file 2).

### Between group change in HrQol, spinal strength and spinal curvature

Comparing change pre/post intervention in the LFG and HFG, there no significant between-group changes in self-image, *p* = 0.473 (Table 3). There were significant between-group changes in spinal extensor strength, *p* = 0.018. There were no significant between-group changes in Timed Loaded standing, *p* = 0.800. Both groups improved equally in kyphometer measured kyphosis, *p* = 0.409. There was significant between-group changes in spinal extensor strength in the age-adjusted model, *p* = 0.036 and borderline between-group change in the age and sex adjusted models, *p* = 0.050.

### Feasibility

Adherence during the 3 month intervention was 83% in the LFG and 79% in the HFG (Table 4). There were no adverse events in either group. 69% (18/26) of the low function participants and 68% (51/76) of the high function participants had non-reportable events including including pain and stiffness several hours to days after exercise, often from a pre-existing musculoskeletal complaint, which resolved within an expected period of time [44]. The LFG reported an average 2.83 events per person and the HFG reported 2.35 events per person.

**Table 4:** Adherence and safety/adverse events in the LFG and HFG

	Low-Function group (LFG) *N* = 26	High-Function group (HFG) *N* = 75
Mean adherence to sessions (percent)	20 (83)	19 (79)
Number of people reporting non-reportable events^a^ (percent)	18 (69)	51 (68)
Number non-reportable events	51	120
Range of non-reportable events per person	1 to 8	1 to 8
Mean number of non-reportable events per person	2.8	2.4
Number of people reporting adverse events^b^	0	0

*LFG* low functioning group, *HFG* high functioning group

^a^Non-reportable events are symptoms that may occur during the intervention and disclosed in the consent, and resolve within an expected duration of time

^b^Adverse events are defined as any untoward or unfavorable medical occurrence

### Sample size calculation

Based on a 2-by-2 repeated measures design a sample size of *n* = 138 (69 in invervention, 69 in control group) achieves 80% power to detect a difference in mean change of 0.6 points on the SPPB at a 0.050 significance level (alpha) using a two-sided, two-sample t-test (Additional file 3).

## Discussion

The primary purpose of this secondary data analysis was to explore the hypothesis that targeting kyphosis will improve physical function in a low functioning cohort of older adults with hyperkyphosis who are transitioning to physical frailty. While there were no statistically significant changes in physical function in the participants with low physical function in the unadjusted analysis, there were significant improvements in SPPB in the adjusted models, and there were clinically significant improvements for the mean SPPB score and gait speed after the targeted kyphosis intervention in the LFG in both unadjusted and adjusted models. There were small improvements in self-image in both groups. Kyphosis improved across both LFG and HFG, even though spinal extensor strength and Time Loaded Standing had relative, negative changes the LFG and relative positive changes in the HFG. Adherence to the intervention, adverse events and non-reportable events were similar in both groups suggesting the intervention is acceptable and safe in a low functioning cohort.

As we hypothesized, SPPB improved a clinically significant amount in the LFG. SPPB changed 0.62 (95%CI: − 0.2 to 1.44) points in the unadjusted model and improved 0.77 (95%CI: 0.23 to 1.3) and 0.84 (95%CI: 0.31 to 1.38) points in the age and age and sex adjusted models in the LFG. This is similar to 2 previous randomized studies that included lower functioning adults [17, 18]. Benedetti et al. [17] performed a targeted exercise program among older adults with hyperkyphosis, and both active and control groups improved SPPB while only the control group improvement was significant (*p* = 0.03). Baseline score for SPPB in the active group was 10.33 (SD = 1.17) points and improved 0.74 points after the intervention, and the control group baseline score was 9.38 (SD = 1.04) points and improved 1.08 points, consistent with the 0.62 (SD = 2.00) point change in our LFG. However, both groups in the Benedetti trial received an exercise program. The active group received targeted spinal strengthening while the control group received a global posture training program, which may explain why both groups improved. Jang et al. [18] tested the efficacy of an 8-week kyphosis correction exercise program in a frailer group than our current LFG. The experimental and control group baseline SPPB scores were 8.7 points and 8.9 points, respectively. The control group received written instructions on the intervention exercises and performed them independently, and the experimental group received in-clinic training and feedback on the exercises. After the intervention period, SPPB scores improved 1.4 points in the experimental group and did not change in the control group. These results support our hypothesis that a combined kyphosis exercise and posture training program in a low functioning cohort may improve physical function, but a larger study powered to detect a significant change in physical function is needed.

It is possible targeting kyphosis in older adults with low physical function and hyperkyphosis may slow expected age-related decline in physical function. The minimum clinically important difference (MCID) has been reported to be between 0.3 to 0.8 points on the SPPB using a combination of both distribution and anchor-based methods in two different studies evaluating cohorts similar to the current cohort (age range 70–89) of mildly frail older adults. This suggests the 0.62 (95%CI: − 0.2 to 1.44) point improvement on the SPPB in the LFG may be clinically relevant [45, 46]. Furthermore older adults in LFG improved from a baseline SPPB of 9.62 (1.2) to a score greater than the cut-off score of 10 suggesting targeting kyphosis may mitigate risk of future disability [22]. The LFG improved gait-speed 0.04 (95%CI: − 0.02 to 0.11) m/s which may be clinically relevant given MCID has been reported to be between 0.03 to 0.06 m/s utilizing both distribution and anchor-based methods [45, 46]. In fact, on average 3 years after the SCOR intervention, kyphosis was maintained and gait speed improved an additional 0.08 m/s, highlighting the potential long-term benefits of a short-term kyphosis intervention [47]. Moreover, Merchant et al. [48] concluded trunk adaptations including flexed posture precede declines in gait speed in a cross-sectional gait kinematics study of older Chinese men who were mildly frail when compared to fit older men. Our LFG may have been transitioning to frailty and improving kyphosis may have associated with a small improvement in gait speed, possibly slowing the progression to frailty.

We investigated change in self-image, which is central to psychological well-being and a measure of HRQoL and has been linked to kyphosis [1, 49]. Self-image, a subdomain of self-esteem, is related to exercise self-efficacy, one’s belief in one’s ability participate in regular exercise [50]. We observed a small within group improvement in self-image for both the LFG 0.19 (95%CI: 0.02 to 0.37) points and the HFG 0.24 (95%CI: 0.14 to 0.34) points after receiving the intervention and both groups responded simarly, *p* = 0.473. These changes are less than the 0.43 (95% CI: 0.24 to 0.61) point change reported after a 6-month targeted kyphosis intervention delivered 3 times a week [15]. Improving self-image through a targeted kyphosis intervention may be useful in facilitating maintence of physical activity in older adults; however, neither LFG or HFG group improved physical activity significantly after the intervention and it is possible that a 3-month intervention was not adequate to facilitate a change. Sinaki et al. conducted a 2 year home-based progressive back strengthening exercise intervention randomized trial in post-menopausal women and found physical activity improved significantly more in the intervention group, *p* = 0.009, at the 2 year mark suggesting a longer exposure to kyphosis targeted intervention may lead to improved physical activity [51].

Spinal extensor strength and endurance decreased in the LFG, but increased in the HFG after the intervention (− 6.0% vs + 6.8%, *p* = 0.018) and (− 8.8 s vs + 5.2 s, *p* = 0.080), respectively. Weakness in the spinal extensors increases risk of kyphosis progression and more recently trunk muscle composition has been linked to increased risk of kyphosis progression and decline in physical function [52–55]. The LFG had lower baseline spinal endurance (112 s vs 138 s) and higher baseline pain scores (3.3 vs 2.0 points) which may have affected their ability to improve during the intervention, and they may have benefited from more time to accommodate to the strengthening intervention provided in the 3 month intervention. There is a dose-response relationship to strength training in older adults and while 12-weeks leads to improved strength, longer durations up to 53 weeks leads to greater improvements in muscular strength [56]. While trunk muscle composition may be an important biologic factor in decline in physical function in older adults, it is unknown if improved trunk muscle composition has a mediating effect on improved physical function. Future trials targeting older adults with hyperkyphosis and low physical function should consider assessing trunk muscle composition in future trials to investigate it as a mediating factor for change in physical function.

Several factors indicate the intervention was well-tolerated in both the low- and high-functioning groups. Adherence to the intervention was similar in both groups. The LFG attended an average of 1 more class than the HFG participants (20 versus 19 of the possible 24 classes) and a similar number of people had non-reportable complaints in the LFG and HFG (69% vs 68%). The LFG started the intervention with higher resting pain scores (3.3 vs 2.0 points); however, both groups had small non-significant reductions in pain over the 3 month intervention period (− 0.3 points in HFG, − 0.2 points in LFG). There were no reportable adverse events in either group. The adherence rates, non-reportable events and reportable adverse events are consistent with findings reported in a meta analysis (*n* = 13 studies) evaluating the impact of exercise on hyperkyphotic posture in post-menopausal women and men [57]. These results suggest testing this intervention in a larger cohort of older adults who have low physical function is feasible and safe. Specific drop-out data within the HFG and LFG is unavailable which could have artificially influenced adherence and safety in either group. However, 9 participants dropped out in the first week due to lack of interest or time and 2 did not have analyzable baseline radiographs which reflects favorably on the integrity of the cohorts.

This analysis suggests that lower functioning older adults who are transitioning to frailty with hyperkyphosis may improve physical function after a targeted kyphosis intervention; however, there were several limitations. This was a post-hoc exploratory analysis of previously reported data from a randomized controlled trial. Causal relationships cannot be established because the number of older adults in the low functioning group was small, and the analysis was not powered to detect significant changes in physical function. Furthermore, we did not adjust for covariates due to the small sample size in our analyses, although we did perform age and sex-adjusted sensitivity analyses, and it is possible the changes in physical function were attributable to other covariates. There were differences in baseline characteristics of BMI, self-reported health, physical function and HrQol in the LFG and HFG that may have influenced results. The results in our LFG are not generalizable to a frail cohort of older adults given participants needed to be able to rise from a chair without their hands and walk a block without an assistive device to meet recruitment criteria. However we can generalize to a cohort of older adults who are transitioning to physical frailty based on a SPPB score of 10 or less. The difference in baseline SPPB scores were small (9.6 vs 11.4) which could limit generalizability to all older adults; however, there was a significant difference between change in SPPB score after controlling for age, thus this intervention may be generalizable to older adults transitioning to frailty regardless of age. Furthermore, for each unit increase in SPPB score, the odds of incident disability risk decrease by approximately 25%, suggesting the small magnitude of difference between the LFG and HFG baseline SPPB scores is clinically relevant [20]. Despite the limitations, the magnitude and direction of changes in SPPB and gait speed suggest further study is warranted to test the hypothesis that lower functioning older adults who are transitioning to frailty with hyperkyphosis who participate in a targeted kyphosis intervention will improve physical function. The strength of this study is that no prior published studies have specifically targeted lower functioning older adults with hyperkyphosis to determine the effects of a kyphosis intervention on physical function. However, our results are consistent with other studies that included a range of physical functioning, including lower functioning older adults with hyperkyphosis [17, 18].

## Conclusion

The results from this secondary analysis suggest that older adults with low physical function who are transitioning to frailty and hyperkyphosis may improve physical function after a targeted kyphosis intervention. Older adults with low physical function in our cohort adhered to and safely participated in a progressive, targeted high-intensity kyphosis exercise and posture training intervention. This observation needs to be confirmed in larger adequately powered studies. Further study is warranted with a large sample of low functioning older adults and hyperkyphosis to confirm these observations and determine if hyperkyphosis is relevant on the causal path towards decline in physical function and frailty.

## Supplementary Information

**Additional file 1:.** Age-adjusted mean change pre/post intervention in outcome measures in the HFG, LFG with confidence intervals (CI).

**Additional file 2:.** Age and sex adjusted mean change pre/post intervention in outcome measures in the HFG, LFG with confidence intervals (CI).

**Additional file 3:.** Numeric Results for Comparing Mean Change in a Repeated Measures Design – Sample size estimation to determine change in Short Physical Performance Battery (SPPB) in a low functioning cohort after a kyphosis intervention.

## Data Availability

The datasets used and/or analysed during the current study are available from the corresponding author on reasonable request.

## Abbreviations

SCOR: Specialized Center of Research Kyphosis randomized trial
HrQol: Health-related Quality of Life
modified PPT: Modified Physical Performance Test
SPPB: Short Physical Performance Battery
LFG: Low function group
HFG: High function group
MCID: Minimum Clinically Important Difference
TUG: Timed Up and Go
6 MW: Six Minute Walk test
PROMIS: Patient-Reported Outcome Measuremement Information System
PASE: Physical Activity Scale for the Elderly
