# Supplementary material for: Secondary analysis of change in physical function after exercise intervention in older adults with hyperkyphosis and low physical function
Source: BMC Geriatr. 2021 Feb 22;21:133. doi: 10.1186/s12877-021-02062-8 (PMC7901174; doi:10.1186/s12877-021-02062-8)
Supplement: Supplementary file 1 — Additional file 1:. Age-adjusted mean change pre/post intervention in outcome measures in the HFG, LFG with confidence intervals (CI). [file 12877_2021_2062_MOESM1_ESM.docx]

Additional file 1. Age-adjusted mean change pre/post intervention in outcome measures in the HFG, LFG with confidence intervals (CI)

|  | **Low -Function group**  **(LFG)** | **High-Function group**  **(HFG)** | **LFG vs HFG*** |
| --- | --- | --- | --- |
|  | Mean difference  (95% CI) | Mean difference  (95% CI) | p-value |
| **Physical Function** | | | |
| Short Physical Performance Battery (SPPB) (0-12 points) | 0.77^**^ (0.23 to 1.3) | -0.09 (-0.40 to 0.22) | 0.008 |
| 4-meter gait speed (m/s) | 0.04^**^ (-0.05 to 0.12) | -0.01 (-0.06 to 0.04) | 0.037 |
| Modified PPT (0-36 points) | -0.2 (-3.0 to 2.6) | -1.3 (-3.0 to 0.3) | 0.496 |
| Time up and go (seconds) | -0.2 (-0.6 to 0.2) | 0.06 (-0.2 to 0.3) | 0.2401 |
| 6 Minute walking test (meters) | 8.9 (-12.6 to 30.4) | 6.3 (-6.0 to 18.6) | 0.838 |
| PROMIS Physical Function t-score (0-100 points) | 0.95 (-1.5 to 3.7) | 2.0 (0.6 to 3.3) | 0.480 |
| **Health-related quality of life** | | | |
| SRS 30 Self-image (0-5 pts) | 0.19 (0.01 to 0.36) | 0.24 (0.14 to 0.34) | 0.582 |
| PROMIS Global Health Scale (0-100 points) | 1.3 (-0.3 to 2.8) | 0.2 (-0.6 to 1.1) | 0.255 |
| PROMIS Global Health scale, Mental Health t-score (0-100 points) | 1.2 (-0.9 to 3.3) | 0.3 (-0.9 to 1.5) | 0.4780 |
| PROMIS Global Health scale, Physical Health t-score (0-100 points) | 2.2 (0.15 to 4.21) | 0.5 (-0.6 to 1.6) | 0.1633 |
| PASE activity (0-793) points | 9.7 (-8.1 to 27.4) | 0.5 (-10.0 to 10.9) | 0.385 |
| **Spinal Strength** | | | |
| Spinal Flexion (percent peak torque/bodyweight) | 0.5 (-2.5 to 3.5) | 1.3 (-0.4 to 3.1) | 0.637 |
| Spinal extensor (percent peak torque/bodyweight) | -5.6 (0.5 to 10.8) | 5.6 (0.50 to 10.8) | 0.037 |
| Time loaded standing (seconds) | -8.7 (-21.5 to 4.0) | 5.1 (-2.1 to 12.5) | 0.069 |
| **Spinal Curvature** | | | |
| Cobb Angle (degrees) | -0.73 (-2.5 to 1.1) | -1.5 (-2.6 to -0.4) | 0.485 |
| Kyphosis (degrees) | -3.5 (-5.8 to -1.1)** | -3.6 (-4.9 to -2.2)** | 0.960 |
| Lordosis (degrees) | 0.7 (-2.1 to 3.3) | -1.6 (-3.1to -0.07) | 0.161 |

CI= confidence interval, Modified PPT=Physical Performance Test, SRS Scoliosis Research Society, PASE= Physical Activity Scale for the Elderly, HFG= high functioning group, LFG= low functioning group, *p values for comparison between LFG and HFG, ^**^ denotes change scores surpassing minimum clinical change estimates (SPPB .03 to .08 points and gait speed .03 to .06 m/s and kyphosis Minimum Detectable Change 2.51 degrees)^15,43,44^
