# Supplementary material for: Secondary analysis of change in physical function after exercise intervention in older adults with hyperkyphosis and low physical function
Source: BMC Geriatr. 2021 Feb 22;21:133. doi: 10.1186/s12877-021-02062-8 (PMC7901174; doi:10.1186/s12877-021-02062-8)
Supplement: Supplementary file 2 — Additional file 2:. Age and sex adjusted mean change pre/post intervention in outcome measures in the HFG, LFG with confidence intervals (CI). [file 12877_2021_2062_MOESM2_ESM.docx]

Additional file 2. Age and sex adjusted mean change pre/post intervention in outcome measures in the HFG, LFG with confidence intervals (CI)

|  | **Low -Function group**  **(LFG)** | **High-Function group**  **(HFG)** | **LFG vs HFG*** |
| --- | --- | --- | --- |
|  | Mean difference  (95% CI) | Mean difference  (95% CI) | p-value |
| **Physical Function** | | | |
| Short Physical Performance Battery (SPPB) (0-12 points) | 0.84^**^ (0.30 to 1.38) | -0.12 (-0.43 to 0.19) | 0.003 |
| 4-meter gait speed (m/s) | 0.04^**^ (-0.04 to 0.13) | -0.01 (-0.06 to 0.04) | 0.278 |
| Modified PPT (0-36 points) | -0.01 (-2.8 to 2.8) | -1.4 (-3.0 to 0.2) | 0.402 |
| Time up and go (seconds) | -0.3 (-0.7 to 0.1) | 0.08 (-0.2 to 0.3) | 0.149 |
| 6 Minute walking test (meters) | 9.9 (-11.8 to 31.7) | 6.0 (-6.4 to 18.3) | 0.758 |
| PROMIS Physical Function t-score (0-100 points) | 0.8 (-1.62 to 3.30) | 2.0 (0.60 to 3.40) | 0.429 |
| **Health-related quality of life** | | | |
| SRS 30 Self-image (0-5 pts) | 0.19 (0.01 to 0.37) | 0.24 (0.14 to 0.34) | 0.632 |
| PROMIS Global Health Scale (0-100 points) | 1.3 (-0.2 to 2.9) | 0.2 (-0.6 to 1.1) | 0.220 |
| PROMIS Global Health scale, Mental Health t-score (0-100 points) | 1.3 (-0.9 to 3.4) | 0.3 (-1.0 to 1.5) | 0.278 |
| PROMIS Global Health scale, Physical Health t-score (0-100 points) | 2.3 (0.3 to 4.4) | 0.4 (-0.7 to 1.6) | 0.131 |
| PASE activity (0-793) points | 10.2 (-7.9 to 28.2) | 0.3 (-10.2 to 10.8) | 0.363 |
| **Spinal Strength** | | | |
| Spinal Flexion (percent peak torque/bodyweight) | 0.5 (-2.6 to 3.6) | 1.3 (-0.4 to 3.1) | 0.667 |
| Spinal extensor (percent peak torque/bodyweight) | -5.2 (-14.3 to 3.9) | 5.5 (0.3 to 10.7) | 0.050 |
| Time loaded standing (seconds) | -9.2 (-22.2 to 3.8) | 5.3 (-2.1 to 12.8) | 0.064 |
| **Spinal Curvature** | | | |
| Cobb Angle (degrees) | -0.7 (-2.5 to 1.2) | -1.5 (-2.6 to -0.4) | 0.442 |
| Kyphosis (degrees) | -3.2^**^ (-5.7 to -0.8) | -3.7^**^ (-5.0 to -2.3) | 0.743 |
| Lordosis (degrees) | 0.5 (-2.2 to 3.3) | -1.5 (-3.0 to -0.02) | 0.632 |

CI= confidence interval, Modified PPT=Physical Performance Test, SRS Scoliosis Research Society, PASE= Physical Activity Scale for the Elderly, HFG= high functioning group, LFG= low functioning group, *p values for comparison between LFG and HFG, ^**^ denotes change scores surpassing minimum clinical change estimates (SPPB .03 to .08 points and gait speed .03 to .06 m/s and kyphosis Minimum Detectable Change 2.51 degrees)^15,43,44^
