# Supplementary material for: Secondary analysis of change in physical function after exercise intervention in older adults with hyperkyphosis and low physical function
Source: BMC Geriatr. 2021 Feb 22;21:133. doi: 10.1186/s12877-021-02062-8 (PMC7901174; doi:10.1186/s12877-021-02062-8)
Supplement: Supplementary file 3 — Additional file 3:. Numeric Results for Comparing Mean Change in a Repeated Measures Design – Sample size estimation to determine change in Short Physical Performance Battery (SPPB) in a low functioning cohort after a kyphosis intervention. [file 12877_2021_2062_MOESM3_ESM.docx]

Additional file 3

Numeric Results for Comparing Mean Change in a Repeated Measures Design – Sample size estimation to determine change in Short Physical Performance Battery (SPPB) in a low functioning cohort after a kyphosis intervention.

| **Target**  **Power** | **Actual**  **Power** | **N1** | **N2** | **N** | **δ** | **σ(T1)** | **σ(T2)** | **ρ** | **σ(Diff)** | **Alpha** |
| --- | --- | --- | --- | --- | --- | --- | --- | --- | --- | --- |
| 0.80 | 0.80373 | 69 | 69 | 138 | 0.6 | 1.0 | 1.3 | 0.400 | 1.285 | 0.050 |
| 0.81 | 0.81490 | 71 | 71 | 142 | 0.6 | 1.0 | 1.3 | 0.400 | 1.285 | 0.050 |
| 0.82 | 0.82028 | 72 | 72 | 144 | 0.6 | 1.0 | 1.3 | 0.400 | 1.285 | 0.050 |
| 0.83 | 0.83062 | 74 | 74 | 148 | 0.6 | 1.0 | 1.3 | 0.400 | 1.285 | 0.050 |
| 0.84 | 0.84045 | 76 | 76 | 152 | 0.6 | 1.0 | 1.3 | 0.400 | 1.285 | 0.050 |
| 0.85 | 0.85425 | 79 | 79 | 158 | 0.6 | 1.0 | 1.3 | 0.400 | 1.285 | 0.050 |
| 0.86 | 0.86286 | 81 | 81 | 162 | 0.6 | 1.0 | 1.3 | 0.400 | 1.285 | 0.050 |
| 0.87 | 0.87101 | 83 | 83 | 166 | 0.6 | 1.0 | 1.3 | 0.400 | 1.285 | 0.050 |
| 0.88 | 0.88242 | 86 | 86 | 172 | 0.6 | 1.0 | 1.3 | 0.400 | 1.285 | 0.050 |
| 0.89 | 0.89292 | 89 | 89 | 178 | 0.6 | 1.0 | 1.3 | 0.400 | 1.285 | 0.050 |
| 0.90 | 0.90257 | 92 | 92 | 184 | 0.6 | 1.0 | 1.3 | 0.400 | 1.285 | 0.050 |

Target Power is the desired power value (or values) entered in the procedure. Power is the probability of

rejecting a false null hypothesis.

Actual Power is the power obtained in this scenario. Because N1 and N2 are discrete, this value is often

(slightly) larger than the target power.

N1 and N2 are the number of items sampled from each population.

N is the total sample size, N1 + N2.

δ is mean change of group 2 minus the mean change of group 1 assuming the alternative hypothesis.

σ(T1) and σ(T2) are the standard deviations of measurements at time 1 and time 2, respectively.

ρ is correlation between a pair of observations made on the same subject. σ(Diff) is the standard deviation of the paired differences, assumed equal for the two groups.

Alpha is the probability of rejecting a true null hypothesis.
